# Activation of NOTCH1 by Shear Force Elicits Immediate Cytokine Expression in Human Chondrocytes

**DOI:** 10.3390/ijms21144958

**Published:** 2020-07-14

**Authors:** Hao-Jen Cheng, Wan-Ting Hsu, Cheng-Nan Chen, Chin Li

**Affiliations:** 1 Department of Biomedical Sciences, National Chung Cheng University, Chiayi 621, Taiwan; md.cheng.1@gmail.com (H.-J.C.); white8020@hotmail.com (W.-T.H.); 2 Department of Orthopedics, Shinnhomei Clinic, Chiayi 600, Taiwan; 3 Department of Biochemical Science and Technology, National Chiayi University, Chiayi 600, Taiwan; cnchen@mail.ncyu.edu.tw

**Keywords:** shear force, NOTCH1, chondrocytes, osteoblasts

## Abstract

Osteoarthritis is caused by overloading of joints and is characterized by inflammation-induced disruption of cartilage structure. Current treatment strategy aims to relieve inflammation and prevent further deterioration of joint function. However, how mechanical force leads to inflammation and deterioration of chondrocyte function still remains incompletely understood. To explore the force-regulated molecular mechanism, an in vitro hydraulic shear force experiment to simulate the condition of force loading was required. The result demonstrated that multiple cytokines and immune regulators, including interleukin 8, interferon β, TRAF1 and TNFAIP3, were significantly increased by shear force within two hours of treatment. Moreover, JAG1 and HES1 were drastically upregulated as well, suggesting that NOTCH1 signaling is activated by shear force. Short-term expression of NOTCH1 intracellular domain activated a similar set of cytokines, indicating that NOTCH1 responds to shear force and activates downstream genes. When incubated under the medium conditioned by NOTCH1-activated chondrocyte, osteoblasts expressed higher levels of interferon β and interferon λ. Together, our results indicated that NOTCH1 functions as a force sensor and promotes expression of cytokines and immune regulators from shear-force bearing chondrocytes.

## 1. Introduction

Osteoarthritis (OA) is the most common age-related joint disease, causing pain and swelling of joints [1]. Multiple factors, including excessive mechanical loading and systematic metabolic abnormality, contribute to development of clinical OA although weighting of each factor may differ between different joints [2,3,4]. Just as the life expectancy of people in developed countries increases, so too does the number of people immobilized by OA [5,6]. Since disabled elders impose heavy burdens on health and social security systems, there is an urgent need to effectively address this medical problem.

At the cellular level, osteoarthritis is the gradual loss of the structure and function of cartilage [1,7]. Under normal conditions, chondrocytes secrete type II collagens and proteoglycans to maintain cartilage matrix [8]. However, during the progression of OA, chondrocytes undergo phenotypic changes through a process of dedifferentiation [9]. The changes include the acquisition of a fibroblast-like morphology, decreases in type II collagen expression, and increases in type I collagen expression [10,11]. This switch of matrix protein expression results in synovial fibrosis, causing joint stiffness and pain. In addition, bone spurs may occur to compensate for the loss of cartilage, although it may not be the main cause of symptoms. How this transformation of cells affects the individual remains incompletely understood.

A molecular hallmark of OA is the elevation of inflammatory cytokines in the synovial microenvironment [12]. Although not all roles of inflammatory cytokines are understood in OA progression, it is clear that elevated inflammatory cytokines play an overall catabolic role in the OA joint. The function of chondrocytes is subsequently altered and unable to compensate for the loss of matrix, resulting in a net loss of cartilage matrix. These inflammatory cytokines include IL-6 and IL-8. The elevated levels of these two cytokines are observed in the synovial fluid as well as in the serum of OA patients [13,14]. In addition, IL-1β and TNFα have been implicated in promoting OA progression [15,16]. Expression of IL-1β and TNFα is activated by NFκB and AP-1 transcription factors and consequently induces autocrine production of other cytokines and mediators, including MMP9, MMP13, and IL-6 [15,17], further exciting inflammation. Furthermore, IL-1β has been demonstrated to inhibit production of cartilage extracellular matrix components, including aggrecan and types II and IX collagen [10]. Breakdown of extracellular matrix can promote inflammation and cartilage loss. In a rabbit animal model, the introduction of fibronectin fragments to the knee joints has led to cartilage damage by inducing inflammatory cytokines TNFα and IL-1β as well as matrix metalloproteinases MMP1 and MMP3 [18,19]. Thus, the damage of cartilage activates inflammation, which in turn reduces the repair ability of chondrocytes, further aggravating cartilage damage.

NOTCH1 signaling is essential for development and stem cell maintenance. During bone and cartilage development, NOTCH1 functions to facilitate differentiation of chondrocytes by activating the expression of SOX9 [20,21]. In normal cartilage, highest level of NOTCH1 expression is localized in the superficial zone, but the region with high NOTCH1 expression shifts to the middle zone in OA cartilage. Evidences have also shown that NOTCH1 is frequently activated in the chondrocytes of OA cartilage [22,23,24,25]. Inhibition of NOTCH1 activation by γ-secretase inhibitor restores the decreased expression of collagen II and suppresses MMP13 expression [26]. Hence, activation of NOTCH1 signaling likely plays an important role in the pathogenesis of OA. Besides regulating matrix formation, NOTCH1 is able to sustain NFκB activation and is associated with increased expression of inflammatory cytokine from synoviocytes and chondrocytes [4,27], hence promoting the progression of OA. Despite of the potential involvement of NOTCH1 in OA progression, the precise function of NOTCH1 in chondrocyte remains to be illustrated.

Here, we reported that fluid shear force induces immediate upregulation of NOTCH1 downstream genes and inflammatory cytokines and chemokines. It was reported that NOTCH1 is a mechanical force sensor in endothelial cells [28]. To explore the role of NOTCH1 in force-induced inflammatory response, we expressed the active form of NOTCH1 and found that NOTCH1 elevates the expression of IL-8 and TNFα, two cytokines promoting joint inflammation. Moreover, conditioned medium conditioned by chondrocytes with active NOTCH1 in turn stimulates inflammatory cytokines and chemokines expression from osteoblasts. Together, our data suggests that the NOTCH1 signaling acts as a sensor to relay mechanic pressure and induce expression of inflammatory cytokines in chondrocytes.

## 2. Results

### 2.1. Identification of Shear Stress Immediate Response Genes in Chondrocytes

**Figure 1:**
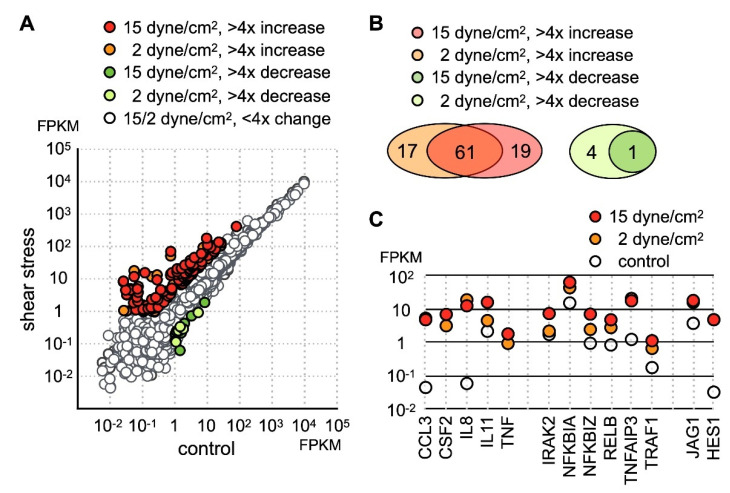
Identification of the fluid shear force stress immediate response genes in SW1353. (**A**) SW1353 cultured on glass plate was subject to low (2 dynes/cm^2^) or high (15 dynes/cm^2^) parallel fluid shear force for 30 min. Expression profiles before and after shear stress was determined by RNA sequencing and expressed as fragments per kilobase of transcript per million mapped reads (FPKM). Genes displaying more than fourfold change were colored. (**B**) 79 and 81 genes are upregulated more than fourfold under low and high shear stress, respectively. In both datasets, 61 genes are found upregulated. The number of genes with more than fourfold downregulation was 4 and 1 under low and high shear stress, respectively. (**C**) The expression level of selected shear stress-activated genes is shown.

To investigate the effect of mechanical loading on the function of chondrocytes, we cultured human chondrocyte cell line SW1353 with low (2 dynes/cm^2^) or high (15 dynes/cm^2^) levels of parallel-direction fluid shear force as the form of shear stress. After stress treatment, the cells were removed from the hydraulic chamber and immediately lysed for RNA collection. Gene expression profiles before and after fluid shear force treatment was determined by RNA sequencing. The sequencing data was mapped to hg19 and estimated for the mRNA level. The RNA level of each gene is expressed as fragments per kilobase of transcript per million mapped reads (FPKM). In this study, genes with FPMK less than 1 in all samples were considered marginally expressed genes and excluded for further analysis. Among the genes with FPKM larger than 1, a significant number of genes displayed an increase in expression level (Figure 1A). In contrast, only 5 genes were downregulated more than fourfold by low fluid shear force, and one of these genes, ZSWIM3, was further downregulated under high fluid shear force.

Among upregulated genes, 80 protein-coding genes exhibited more than fourfold increases under shear stress (Table S2). Among these highly activated genes, 61 genes were upregulated under both low and high shear stress (Figure 1B), while 19 genes were upregulated above fourfold only in the SW1353 cells under high stress. GO enrichment analysis indicated the molecular functions of these upregulated genes were cytokine signaling and transcription regulation. Thus, our data indicated the main cellular response of chondrocytes to fluid shear force stress was the activation of the inflammatory response.

Close inspection of the dataset revealed that a number of genes participating in the inflammatory process included several inflammatory cytokines and chemokines (Figure 1C). Under static condition, IL-8 and CCL3 [29] were expressed at extremely low levels, but the expression level of both genes increased nearly a hundredfold under 2 h of shear stress. In addition to IL-8 and CCL3, TNFα and CSF2 were completely undetectable in untreated cells and were increased significantly after application of shear stress. Correlated with an increase of TNFα expression, the level of TNFAIP3 [30] and TRAF1 [31] was drastically increased as well (Figure 1C). Both TRAF1 and TNFAIP3 were previously identified as osteoarthritis-associated markers. Coupled with an increase of TNFα expression, our data suggested that shear stress activated TNF signaling. TNFAIP3 functions to inhibit NFκB activation and TNFα-mediated apoptosis [32,33]. Incidentally, there was an increase in the expression of NFκB inhibitors NFKB1A and NFKB1Z (Figure 1C) [34].

### 2.2. Induction of Inflammatory Cytokines by NOTCH1

**Figure 2:**
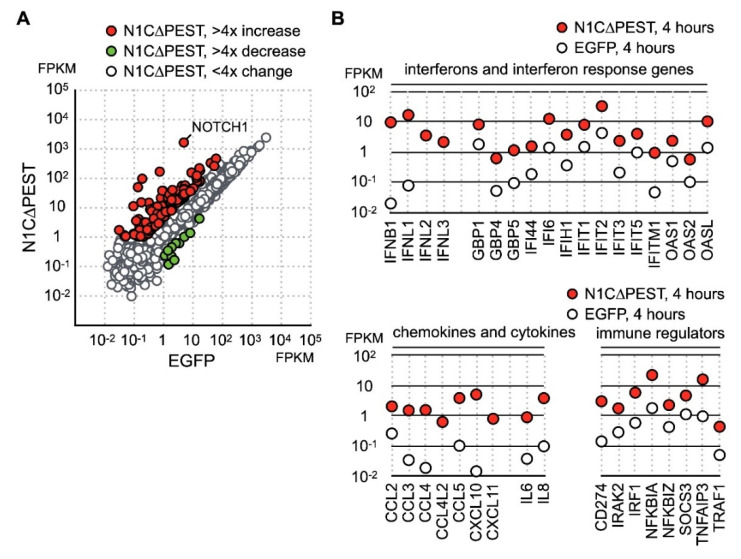
Identification of NOTCH1 activated genes in SW1353. (**A**) SW1353 was transfected with pEGFP-C1 and pEGFP-N1C∆PEST for 2 h and incubated in fresh medium for an additional 2 h. The cells were harvested for analyzation for gene expression by RNA sequencing. Genes with more than fourfold increase and decrease are colored red and green, respectively. Genes with less than fourfold change are colored gray. (**B**) NOTCH1-induced genes are grouped according to their functions as interferons, interferon response genes, cytokines, chemokines, and immune regulators.

Besides the gene function in immune regulation, we also found an increase of JAG1 and HES1 expression (Figure 1C). Given that JAG1 is the ligand for the NOTCH1 receptor and HES1 is the downstream target of activated NOTCH1 signaling, our data indicated that NOTCH1 signaling was activated by fluid shear force. Previous reports have shown that activation of NOTCH1 is associated with production of inflammatory cytokines. However, it is not clear whether activation of NOTCH1 directly induces expression of inflammatory cytokines in chondrocytes. To explore this possibility, we created an EGFP-NOTCH1 PEST domain-deleted intracellular domain (N1C∆PEST) construct and transiently expressed the EGFP-N1C∆PEST in SW1353 to simulate activation of NOTCH1. The change in the expression profile of SW1353 was analyzed by RNA sequencing. For this experiment, we allowed 2 h of transfection time and an additional 2 h of incubation before lysis of the cells for subsequent expression profiling analysis. Given the short period between initiation of transfection and sample collection, it was reasonable to assume the majority of genes with expression level change were those genes immediately responding to the activated NOTCH1 signaling. Similarly, we considered those genes with FPKM smaller than one in any specimens as marginally expressed and excluded those genes in subsequent analysis. The sequencing result showed that 139 genes displayed a fourfold increase and 6 displayed a decrease in SW1353 after 4 h of simulated NOTCH1 activation (Figure 2A and Table S3). Ontological analysis of these upregulated genes showed that NOTCH1 signaling induced expression of cytokines, interferons, interferon response genes, chemokines, and immune response regulators.

Cross referencing the NOTCH1 activated genes with the fluid shear force stress dataset showed that many of fluid shear force stress activated genes also respond to NOTCH1 signaling. These genes included cytokines IL-8 and TNF as well as NFκB signaling regulators NFKB1A, NFKB1Z, TNFAIP3, and TRAF1 (Figure 2B). This observation implied that activation of NOTCH1 is an immediate event of fluid shear force stress treatment, and activated NOTCH1 signaling in turn induces the expression of cytokines and immune regulators. Our data, hence, indicates that NOTCH1 functions to elicit inflammatory response in mechanical force loaded cartilages. In addition, we observed a drastic increase of type III interferons, including IFNL1, IFNL2, and IFNL3 (Figure 2B). Expression of many known interferon response genes was also increased in correlation with an increase in interferon production (Figure 2B). Besides cytokines, genes participating in immune response regulation were also upregulated. The most notable gene among these NOTCH1-activated genes was PD-L1, encoded by CD274. PD-L1 is a target of anti-cancer immunotherapy, but its role in OA is not clear.

**Figure 3:**
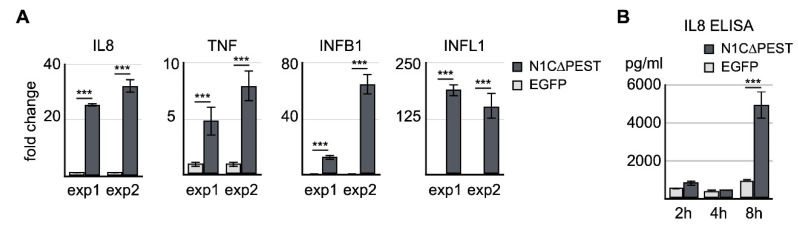
Confirmation of NOTCH1-activated genes by qRT-PCR and ELISA. (**A**) EGFP and EGFP-N1C∆PEST was transiently expressed in SW1353. Four hours after initiation of transfection, total RNA was extracted and analyzed for the expression levels of IL-8, TNF, IFNB1, and IFNL1 by qRT-PCR. Two independent experiments were carried out. The symbol of three asterisks indicates *p*-value smaller than 0.001. (**B**) Culture medium was collected 2 h, 4 h, and 8 h after transfection. The level of IL-8 in the medium was determined by ELISA.

To confirm the result of RNA sequencing, we carried out independent transfection experiments and examined the expression levels of IL-8, TNF, IFNB1, and IFNL1 by quantitative RT-PCR. Our result of qRT-PCR was consistent with the sequencing data indicating that there was a significant increase of IL-8, IFNB1, and IFNL1 (Figure 3A). We also assayed the level of IL-8 in the cultured medium of transfected cells by ELISA. The result showed that the IL-8 protein level in the culture was drastically increased 8 h after expression of active NOTCH1 (Figure 3B). Thus, our data indicates the fluid shear force stress-induced inflammatory reaction is at least in part mediated by activation of the NOTCH1 signaling.

### 2.3. Effect of NOTCH1-Induced Cytokines on Osteoblast

A common symptom of OA is growth of bone spurs. One possibility is that NOTCH1-induced cytokines from chondrocytes subsequently affect the function of osteoblasts. To explore this possibility, we transfected SW1353 with either EGFP or N1C∆PEST for 2 h. The medium containing the transfection reagent was then removed and replaced with fresh medium. After another 2 h, the medium was collected and used to culture osteoblast cell line hFOB 1.19 in a 1:1 ratio with fresh medium. At 4 and 24 h after incubation in the conditional medium, the cells were harvested and the expression profiles were determined by RNA sequencing.

**Figure 4:**
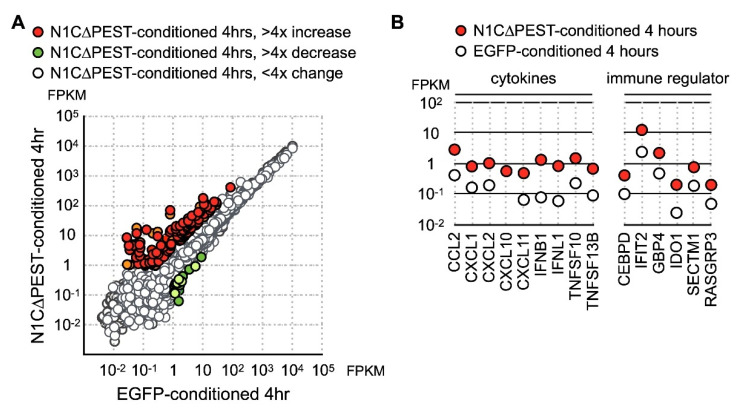
Upregulation of cytokine and chemokine in osteoblast cell line hFOB by conditional medium from N1C∆PEST-transfected SW1353. SW1353 was transfected with pEGFP-C1 or pEGFP-N1C∆PEST for 2 h and continued incubation in fresh medium for another 2 h. The culture medium was harvested and mixed with fresh medium in a 1:1 ratio. The osteoblast cell line hFOB was subsequently cultured in the conditioned medium. At 0 and 4 h under conditioned medium, RNA was collected from hFOB and analyzed by RNA sequencing. (**A**) At four hours under conditioned medium, the expression profile of hFOB was analyzed by RNA sequencing. Genes with more than fourfold increase and decrease are colored red and green, respectively. Genes with less than fourfold change are colored gray. (**B**) Conditioned medium-induced genes are grouped according to their functions as chemokines, cytokines, and immune regulators.

At four hours under conditioned medium, in the hFOB 1.19 cells treated with EGFP-conditioned medium, nine genes showed more than fourfold increase while nine genes showed more than fourfold decrease. In contrast, hFOB 1.19 incubated in N1C∆PEST-conditioned media had 28 upregulated genes and five downregulated genes with greater than fourfold changes (Figure 4A). Examination of the data showed that these genes induced by N1C∆PEST-conditioned media function in inflammation and immune regulation (Figure 4B).

**Figure 5:**
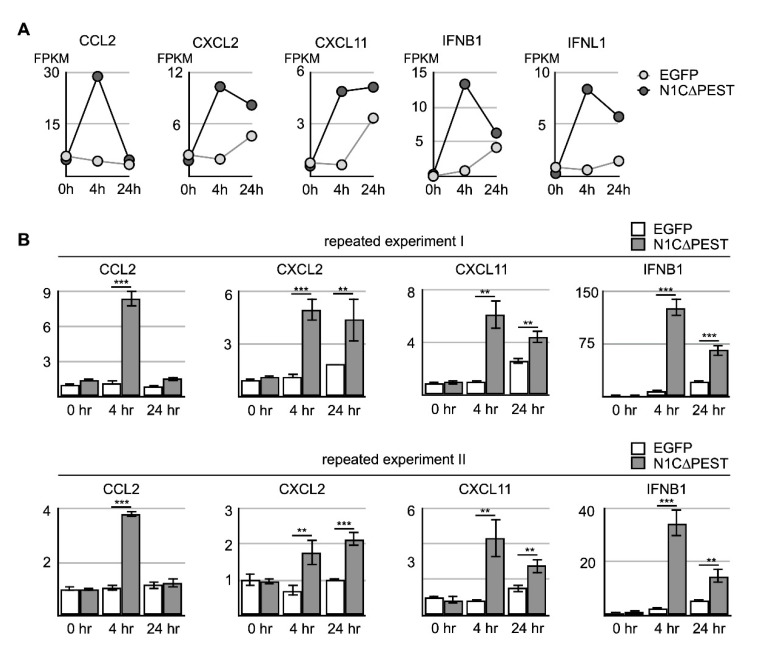
Activation of cytokine expression in osteoblast cell line hFOB by N1C∆PEST-transfected SW1353 conditioned medium. SW1353 was transfected with pEGFP-C1 or pEGFP-N1C∆PEST for 2 h and continued incubation in fresh medium for another 2 h. The culture medium was harvested and mixed with fresh medium in a 1:1 ratio. The osteoblast cell line hFOB was subsequently cultured in the conditioned medium. (**A**) After 0, 4, and 24 h, RNA was collected from conditioned hFOB and analyzed by RNA sequencing. The FPKM of CCL2, CXCL2, CXCL11, IFNB1, and IFNL1 in EGFP-conditioned and N1C∆PEST-conditioned hFOB at 0, 4, and 24 h was shown. (**B**) Two additional independent experiments were carried out. The expression level of CCL2, CXCL2, CXCL11, and IFNB1 was determined by qRT-PCR. GAPDH served as the control. The symbol of two and three asterisks indicates *p*-value smaller than 0.01 and 0.001, respectively.

Analysis of the RNA sequencing data indicated that the level of induced genes started to decline during extended incubation under conditioned medium. Specifically, CCL2 returned to the ground level of untreated cells by 24 h. Although not completely returning to untreated level, the level of CXCL2, IFNB1, and INFL1 nevertheless showed significant decline (Figure 5A). On the other hand, CXCL11 remained at a similar level although the expression level in hFOB treated with the EGFP-conditioned medium also increased somewhat after extended culturing (Figure 5A). To confirm the result of RNA sequencing, we carried out independent experiments and confirmed the expression change of CXCL2, CXCL11, IFNB1, and IFNL1 by qRT-PCR (Figure 5B). Our results indicated that the chondrocyte-released cytokines imposed a short-term stimulating effect on neighboring osteoblasts and led to further accumulation of inflammatory cytokines in the surrounding environments.

## 3. Discussion

During their lifetime, cartilage chondrocytes are constantly subject to mechanical stress, and excess loading leads to inflammation of synovial joint tissues. Repeated chronic inflammation results in an elevated accumulation of cytokines and slowly deteriorates the cartilage’s functions. Previous studies demonstrated that NOTCH1 is activated in OA cartilage chondrocytes [23,24]. In this study, we used a human chondrocyte cell line SW1353 to identify fluid shear force stress-immediate response genes. Upon applying shear stress, the NOTCH1 ligand JAG1 and downstream target gene HES1 was rapidly and drastically upregulated, providing independent experimental evidence that NOTCH1 is activated in chondrocytes by mechanical force. NOTCH1 has been shown to function as a mechanical force sensor in endothelial cells [28]. Together with our data, these findings indicate that NOTCH1 functions as a mechanical force sensor and signal transducer in the stress-bearing tissues.

Although NOTCH1 may function as a mechanical force sensor, it is reasonable to expect that the activation force and response genes are different for cells of distinct origins and functions. For aorta endothelial cells, the NOTCH1 sensor is activated with at least 10 dynes/cm^2^ fluid shear force, but over 20 dynes/cm^2^ is required for effective activation of NOTCH1 in stressed endothelial cells. In comparison, the strength of fluid shear force stress used in our study was 2 and 15 dynes/cm^2^ and the treatment time was 2 h, both much shorter in comparison with the 24 h treatment to activate NOTCH1 in aorta endothelial cells. Within the time period of our treatment, either low or high fluid shear force achieved similar levels of JAG1 and HES1 expression. Given that 2 dynes/cm^2^ shear force simulates normal force received by cartilage, we speculate that the NOTCH1 sensor is activated with a lower threshold in the chondrocytes than in the aorta endothelial cells.

Our data also showed that the gene sets induced by low or high fluid shear force are largely the same, and the induction level for many of individual genes is comparable as well. It appears that both low and high fluid shear force achieves similar cellular effect. Hence, NOTCH1 possibly acts as an on/off switch for sensing mechanical force bearing. On the other hand, the mechanical force imposed on cartilage is more likely in intervals and may even be completely relieved under the rest condition. With the low activation threshold and similar activation level, it is plausible that, rather than a force strength sensor, the NOTCH1 sensor serves as a force-bearing frequency counter.

In response to the fluid shear force, aorta endothelial cells increased the expression of junction proteins, hence forming an even stronger barrier. Similarly, NOTCH1 activation should modulate the chondrocytes to provide the required response to the shear stress. Our data showed that expression of N1C∆PEST in SW1353 led to upregulation of chemokines and interferons, indicating that activation of the NOTCH1 sensor immediately elicits an immune response. Although it remains to be determined how the role of the immediate inflammatory response in cartilage functions, early studies have established that interferon lambda exerts protective effects by suppressing neutrophil infiltration and interleukin 1β expression [35,36]. On the other hand, IL6 and IL-8 may play a pathologic role [37,38]. However, the ELISA assay showed that a significant increase of IL-8 protein was not detected until longer expression of N1C∆PEST, indicating a delay of IL-8 protein expression. Thus, it is likely that the NOTCH1 sensor acts to protect cartilage integrity and functions but may lead to an accumulation of inflammatory cytokines if repeatedly activated. Previous studies showing suppression of NOTCH1 potentially attenuated osteoarthritis provide additional support to this notion [26,39].

Our conditioned medium experiment indicates that chondrocytes with active NOTCH1 could induce inflammatory cytokine from neighboring osteoblast cells. However, in our experiment, the time point to collect the conditioned medium was at 4 h after transfection. At this time point, the IL8 RNA level already shows significant increase but IL8 protein in the medium remains unchanged. Hence, there is a limited amount of SWS1353-released cytokines or chemokines in the conditioned medium. However, it is possible that the limited increase of cytokines or chemokines could still stimulate inflammatory cytokine induction in osteoblast cells. Alternatively, conditioned medium would contain the exosome released from transfected SW1353 sine the centrifugation force used in the preparation process of conditioned medium was not strong enough to remove the exosome. As such, the effect we observed can be also derived from the exosome in the medium. Further investigation is required to explore the postulation that the exosome from force-bearing chondrocytes plays a role in OA.

Fluid shear force immediately increases expression of cytokines, including IL-8, interferon beta, and interferon lambda, as well as HES1 and JAG1. Expression of active NOTCH1 intracellular domain leads to chondrocyte-conditioned medium, inducing higher levels of interferon beta and interferon lambda from osteoblasts. Thus, shear force activates chondrocytes to produce cytokines and chemokines. Our finding may provide a reasonable explanation to previous studies with seemly conflicting interpretation on whether NOTCH1 activation provides protective effects or exacerbates the progression of OA. It is likely transient activation of NOTCH1 by shear force is protective to the functions of chondrocytes. However, under repeated or long-term stress, the NOTCH1 pathway induces release of inflammatory cytokines. The inflammatory cytokines further stimulate the expression of cytokines from osteoblasts, leading to an accumulation of specific cytokines in the microenvironment and attracting inflammatory immune effector cells toward the force-bearing cartilage. This escalation of inflammatory response eventually leads to impeded cartilage repair and results in OA. Our data, hence, indicates that NOTCH1 plays a pivotal role in the initiation of OA.

## 4. Materials and Methods

### 4.1. Cell Culture and Treatment

Human osteoblast cell line hFOB 1.19 was purchased from Bioresource Collection and Research Center, Hsinchu, Taiwan. SW1353 and hFOB 1.19 cell lines were cultured in DMEM and DMEM/F12 medium supplemented with 10% fetal bovine serum, respectively. The protocol to apply shear force on cultured cells was previously described [40]. Briefly, SW1353 was cultured on glass slides for 24 h before shear-stress treatment. The slides were then mounted in a parallel-plate flow chamber connected to a perfusion loop system and were maintained at 37 °C and 5% CO_2_ in a controlled enclosure. The fluid shear stress (τ) generated on the cells by flow was estimated to be 2 and 15 dyn/cm^2^ by using the formula τ = 6 µQ/wh^2^, where μ is the dynamic viscosity of the perfusate, Q is the flow rate, and h and w are the channel height and width, respectively. After 2 h of stress treatment, the cells were lysed immediately in TRIZOL reagent (Thermo Fisher, Waltham, MA, USA) for total RNA extraction.

Expression of active NOTCH1 intracellular domain was as follows: The coding sequence of a PEST domain-deleted intracellular domain of NOTCH1 (N1C∆PEST) was a gift from Dr. Tseng, MJ, Department of Biomedical Sciences, National Chung Cheng University. The sequence was cloned to pEGFP-C1 expression vector to create pEGFP-N1C∆PEST. Introduction of pEGFP-N1C∆PEST and parental PEGFP-C1 into SW1353 was achieved using Lipofectamine 3000 reagent (Thermo Fisher Scientific, Waltham, MA USA) by following the manufacturer-recommended protocol. After two hours of incubation with the transfection reagent, the cells were washed extensively to remove residue transfection reagent and incubated for additional time as indicated.

For conditional medium treatment, SW1353 was transfected with either pEGFP-C1 or pEGFP-N1C∆PEST for two hours and incubated for an additional two hours. The culture medium was then collected and centrifuged to remove insoluble debris. The medium was mixed with fresh medium at a 1:1 ratio and used to culture hFOB 1.19 for the necessary length of time. At each time point, the hFOB cells were lysed in TRIZOL reagent for RNA extraction and subsequent analysis.

### 4.2. RNA Sequencing

For expression profiling, the integrity and concentration of purified RNA samples was determined by capillary electrophoresis and fluorometric quantification. The sequencing library was prepared using SureSelect Strand-Specific RNA Library Prep Kit (Agilent, Santa Clara, CA, USA). Sequencing was carried out on a MiSeq sequencer (Illumina, San Diego, CA, USA). Mapping and annotation of the human genome (hg19) and estimation of gene expression were performed using CLC Genomic Workbench v. 20.0.2 package (Qiagen, Venlo, Netherlands).

### 4.3. Quantitative RT-PCR (qRT-PCR)

Total RNA was purified from cultured cells using TRIZOL reagent (Thermo Fisher) according to the manufacturer’s protocol. The cDNAs were subsequently prepared from the total RNA using MMLV high-performance reverse transcriptase (Illumina) and oligo(dT) as the primer. The condition for PCR detection was 32 cycles of amplification of the template by denaturing at 94 °C for one minute, primer annealing at 55 °C for 30 s, and product extension at 72 °C for 1 min. Quantitative RT-PCR was performed using GoTag qPCR master mix (Promega, Madison, WI, USA) in a MiniOpticon Real-Time PCR System (Bio-Rad Laboratories, Hercules, CA, USA). The condition for 40 cycles of amplification was template denaturing at 94 °C for one minute, primer annealing at 55 °C for 30 s, and product extension at 72 °C for 45 s. The oligomers used in this study are listed in the supplemental material (Table S1). Student’s t-test was carried out to determine the statistical significance between control and experimental groups.

### 4.4. Enzyme-Linked Immunosorbent Assay (ELISA)

The concentration of IL-8 was quantified by ELISA (Abcam, Cambridge, UK). Briefly, the assay plate was coated with the capture antibody at 4 °C overnight. The assay plate was then washed and incubated with blocking buffer at room temperature for 2 h. After washing, specimens and standard antigens were then added to the plate with the detection antibody and incubated at room temperature for 1 h. For detection, streptavidin-conjugated horseradish peroxidase was added to the assay plate after removal of the specimens and additional wash. After 30 min incubation at room temperature and final wash, the TMB substrate was added to the reaction and incubated in the dark for the required development time. The chromogenic reaction was stopped by adding 1 M sulfuric acid solution. The absorption at 450 nm of the reaction solution was measured and used to calculate the standard curve and the concentration of IL-8. Student’s t-test was carried out to determine the statistical significance between control and experimental groups.

### 4.5. Availability of Supporting Data

The sequencing data was deposited in the Sequence Read Archive (SRA), National Center for Biotechnology Information, USA. The BioProject ID is PRJNA630089, and the BioSample accession numbers are SAMN14823957, SAMN14823958, SAMN14823959, SAMN14823960, and SAMN14823961.

## Supplementary Materials

The following are available online at https://www.mdpi.com/1422-0067/21/14/4958/s1, Table S1: The sequences of the oligomers used in this study, Table S2: Protein coding genes drastically upregulated in shear stress-treated SW1353, Table S3: Protein coding genes with more than four changes in expression in N1C∆PEST-expressing SW1353.
