# Supplementary material for: Activation of NOTCH1 by Shear Force Elicits Immediate Cytokine Expression in Human Chondrocytes
Source: Int J Mol Sci. 2020 Jul 14;21(14):4958. doi: 10.3390/ijms21144958 (PMC7404062; doi:10.3390/ijms21144958)
Supplement: Supplementary file 1 [file ijms-21-04958-s001.pdf]

Supplementary Materials

Table S1. The sequences of the primers used in this study.

Gene	Primer Direction	Primer Sequence Used in qRT-PCR
CCL2	forward	5'- CAGCAGCAAGTGTCCCAAAGAAG
	reverse	5'- AGATCTCCTTGGCCACAATGGTC
CXCL2	forward	5'- GGTTAAGAAAATCATCGAAAAGATGCTG
	reverse	5'- CAATAAGCTTCCTCCTTCCTTCTGGT
CXCL11	forward	5'- ACTCCTTCCAAGAAGAGCAGCA
	reverse	5'- AGCCATGCCCTTCACACTCA
IFNB1	forward	5'- TATTGTTGAGAACCTCCTGGCTAATGTC
	reverse	5'- CTCCAGTTTTTCTTCCAGGACTGTCTTC
IFNL1	forward	5'- ACTCCTTCCAAGAAGAGCAGCA
	reverse	5'- AGCCATGCCCTTCACACTCA
IL8	forward	5'- TGCCAGCTGTGTTGGTAGTG
	reverse	5'- GACTGTGGAGTTTTGGCTGTTT
TNF	forward	5'- CCTCTTCAAGGGCCAAGGCT
	reverse	5'- GTCTGGTAGGAGACGGCGAT
GAPDH	forward	5'- CGACAGTCAGCCGCATCTTC
	reverse	5'- CCCAATACGACCAAATCCGTTGA

Table S2. Protein coding genes drastically upregulated in shear stress treated SW1353.

	RPKM			TPM		
Gene	Static	Dyn2	Dyn15	Static	Dyn2	Dyn15
ANKRD1	82.62	243.06	430.85	376.55	1096.55	1991.94
ARC	0.10	1.34	1.10	0.45	6.03	5.11
ATF3	0.30	13.70	9.82	1.35	61.82	45.39
BDKRB1	0.38	0.99	1.94	1.75	4.46	8.97
BHLHE40	27.73	111.82	134.12	126.39	504.48	620.08
CCL3	0.09	5.19	4.57	0.40	23.43	21.15
CCRN4L	5.04	20.39	21.53	22.99	92.00	99.54
CGB7	0.29	0.24	1.24	1.33	1.10	5.72
CSF2	0.08	3.03	6.34	0.38	13.65	29.29
CSRNP1	3.11	38.58	31.18	14.15	174.04	144.14
CTGF	10.17	183.77	184.13	46.36	829.06	851.28

DLC1	3.93	14.55	18.28	17.90	65.62	84.51
DLX2	0.33	1.40	2.34	1.52	6.30	10.81
DUSP1	11.65	111.55	74.20	53.11	503.26	343.04
DUSP2	0.03	3.34	3.25	0.16	15.08	15.04
DUSP5	6.77	57.31	62.24	30.87	258.56	287.76
DUSP6	1.16	13.43	14.03	5.27	60.58	64.86
EDN1	3.97	30.07	28.82	18.08	135.66	133.24
EGR1	0.82	52.88	13.24	3.74	238.55	61.23
EGR2	0.13	16.17	16.21	0.57	72.95	74.95
EGR3	0.03	8.39	8.84	0.12	37.87	40.89
EID3	0.27	1.74	1.51	1.23	7.85	6.96
ERRFI1	10.64	81.74	114.39	48.50	368.76	528.87
FAM43A	1.35	10.56	9.34	6.14	47.65	43.16
FOS	0.22	12.13	2.49	1.02	54.72	11.49
FOSB	0.06	34.41	25.65	0.27	155.26	118.58
FOSL1	23.64	126.73	141.90	107.75	571.75	656.02
FOXC2	4.67	16.02	26.33	21.30	72.25	121.73
GADD45B	16.95	67.68	72.25	77.24	305.34	334.02
GDF15	2.28	11.98	14.92	10.39	54.04	68.99
HBEGF	4.12	24.06	47.50	18.76	108.56	219.61
HES1	0.03	4.42	4.54	0.15	19.96	21.01
ID1	13.51	111.17	97.49	61.56	501.55	450.74
ID4	0.90	5.81	5.55	4.11	26.22	25.66
IFI30	0.05	2.20	2.00	0.24	9.91	9.24
IL11	2.10	4.48	15.93	9.59	20.21	73.65
IL8	0.06	18.50	12.53	0.26	83.44	57.93
INHBA	2.35	6.48	11.36	10.72	29.24	52.51
IRAK2	1.78	2.19	7.30	8.13	9.90	33.73
IRS2	2.24	13.07	11.65	10.20	58.96	53.85
ITPRIP	10.72	43.52	54.12	48.84	196.36	250.21
JAG1	3.70	15.76	17.48	16.86	71.10	80.82
JUNB	14.67	164.46	163.83	66.88	741.97	757.44
KDM6B	2.35	5.54	12.35	10.71	25.00	57.09
KLF10	11.69	71.00	91.87	53.27	320.33	424.72
KLF4	0.47	2.14	2.01	2.13	9.67	9.29

KRTAP1-5	0.06	1.41	1.78	0.26	6.36	8.23
KRTAP2-3	0.08	6.27	3.89	0.35	28.30	17.99
LIF	0.72	14.43	72.71	3.26	65.11	336.16
MAFF	1.24	20.06	20.83	5.67	90.49	96.30
MNT	1.77	6.15	7.04	8.04	27.75	32.54
MYC	6.60	35.90	28.08	30.07	161.96	129.83
NAB2	7.27	18.28	34.81	33.12	82.48	160.93
NFATC2	0.07	0.46	1.00	0.33	2.06	4.64
NR4A1	0.12	0.56	1.53	0.54	2.53	7.10
NR4A2	0.37	4.33	4.74	1.67	19.54	21.90
NR4A3	0.07	3.35	4.98	0.33	15.11	23.04
NUAK2	1.57	6.91	6.10	7.17	31.16	28.21
PER1	2.31	13.78	9.60	10.54	62.18	44.36
PER2	0.73	3.47	3.56	3.33	15.66	16.45
PLK3	3.06	13.23	20.88	13.94	59.69	96.54
PPP1R15A	18.23	100.84	86.37	83.07	454.92	399.32
RELB	0.83	2.74	4.62	3.78	12.34	21.35
SAMD4A	2.91	5.43	11.72	13.27	24.49	54.19
SIK1	5.56	22.44	46.06	25.34	101.24	212.94
SKIL	3.09	7.31	17.74	14.09	32.97	82.03
SLC19A2	2.75	6.07	11.95	12.55	27.37	55.25
SMAD7	2.04	17.79	27.59	9.30	80.28	127.55
SOCS1	0.16	1.21	0.81	0.74	5.45	3.74
SPRY2	1.63	18.35	21.85	7.42	82.80	101.03
SPRY4	0.89	14.38	16.08	4.06	64.90	74.34
STX1A	1.86	4.40	7.45	8.48	19.83	34.43
TCTEX1D4	0.16	1.55	1.35	0.71	6.97	6.24
THBS1	44.28	131.20	172.77	201.78	591.91	798.75
TMEM88	0.08	1.35	2.02	0.34	6.11	9.32
TNFAIP3	1.24	20.11	17.16	5.64	90.72	79.34
TRAF1	0.17	0.63	1.08	0.79	2.86	4.98
TRIB1	1.65	10.55	15.10	7.50	47.60	69.83
ZC3H12A	3.80	27.01	25.53	17.30	121.84	118.05
ZNF469	2.81	3.70	12.68	12.79	16.67	58.61

FPKM: Reads Per Kilobase per Million
TPM: Transcripts Per Million

Table S3. Protein coding genes with more than four change in expression in N1CΔPEST-expressing SW1353.

	**RPKM**		TPM	
**Gene**	**EGFP**	**N1CΔPEST**	**EGFP**	**N1CΔPEST**
ABCC9	0.13	1.04	0.55	4.33
ADAMTSL4	0.51	2.17	2.19	9.04
ADAP1	3.53	18.07	15.29	75.42
AIM2	0.35	2.43	1.50	10.16
APOL4	0.07	1.27	0.31	5.30
ARC	0.21	5.38	0.89	22.45
ATF3	20.01	131.01	86.70	546.88
BATF2	14.08	63.11	61.01	263.45
BBC3	3.61	25.08	15.63	104.70
BEST3	0.19	1.33	0.84	5.55
BIRC3	0.19	1.73	0.81	7.21
BST2	5.49	36.00	23.78	150.28
C3	0.80	4.64	3.46	19.36
C4B	0.36	1.57	1.58	6.56
CACNA1I	0.18	1.29	0.76	5.39
CCL2	2.09	20.52	9.04	85.65
CCL3	1.14	38.56	4.96	160.96
CCL3L1	0.22	13.88	0.93	57.94
CCL4	0.35	22.19	1.52	92.63
CCL4L1	0.13	11.74	0.58	49.01
CCL5	1.47	56.85	6.35	237.31
CCL7	0.44	4.13	1.91	17.24
CCL8	0.90	17.42	3.92	72.71
CD274	7.91	60.53	34.26	252.68
CDR1	20.03	4.34	86.80	18.11
CEMP1	0.00	2.48	0.00	10.34
CFB_1	0.20	1.36	0.86	5.67
CFB_2	0.02	4.78	0.08	19.94
CIITA	0.65	3.54	2.84	14.78
CMPK2	4.30	17.18	18.62	71.73
CSF2	0.08	1.06	0.35	4.42

CTSS	1.14	6.94	4.93	28.97
CXCL10	2.86	131.79	12.40	550.13
CXCL11	1.19	37.32	5.18	155.77
CXCL9	0.14	7.53	0.60	31.44
CYP2J2	0.25	1.52	1.10	6.36
DHX58	5.11	24.07	22.13	100.48
EGR1	12.37	179.80	53.59	750.56
EGR3	0.23	1.04	1.02	4.35
FAM71A	0.05	1.49	0.24	6.23
FOS	2.13	27.98	9.24	116.80
FOXD1	11.16	1.87	48.36	7.79
GBP1	67.28	275.45	291.55	1149.81
GBP2	2.09	10.28	9.05	42.89
GBP4	6.15	35.77	26.64	149.31
GBP5	3.44	27.38	14.92	114.31
GBP6	0.85	3.58	3.67	14.94
GCA	0.52	3.57	2.25	14.92
GCH1	9.95	70.90	43.14	295.94
GNRH2	0.15	1.21	0.67	5.05
GOLGA8N	1.43	0.13	6.18	0.54
HAPLN3	0.88	4.37	3.81	18.26
HIST1H2BE	0.25	1.17	1.10	4.87
HIST1H4C	0.15	1.40	0.66	5.84
HIST2H2AA3	0.71	7.74	3.10	32.33
HIST2H3A	0.31	2.30	1.33	9.61
HIST2H3C	0.23	2.55	0.99	10.66
HIST2H4A	0.94	3.88	4.09	16.20
HIST2H4B	0.58	4.39	2.50	18.33
HLA-DOB	0.17	4.77	0.74	19.93
HLA-DRA	0.35	1.55	1.50	6.49
HLA-F	3.20	13.18	13.88	55.02
HOXB8	0.48	1.92	2.08	8.03
HRASLS2	0.51	3.02	2.22	12.59
HSD17B14	0.40	2.78	1.71	11.61
HSH2D	0.58	4.41	2.50	18.43

ICAM1	0.05	1.25	0.22	5.23
IDO1	2.60	31.11	11.27	129.87
IER3	0.09	5.26	0.40	21.94
IFI27	1.78	8.51	7.72	35.54
IFI30	0.84	6.45	3.65	26.92
IFI44L	1.62	8.89	7.02	37.10
IFIT1B	0.03	1.18	0.14	4.91
IFITM1	18.76	95.71	81.29	399.51
IFNB1	0.60	109.33	2.61	456.40
IFNL1	1.18	198.76	5.12	829.68
IFNL2	0.26	22.83	1.12	95.30
IFNL3	0.19	14.53	0.84	60.66
IL10RA	2.03	9.60	8.80	40.07
IL18BP	0.83	3.97	3.62	16.57
IL1RN	0.58	3.00	2.53	12.54
IL32	0.19	3.71	0.84	15.50
IL4I1	1.21	9.80	5.25	40.90
IL6	0.49	15.44	2.13	64.45
IL8	0.53	34.08	2.29	142.27
IRAK2	2.98	24.75	12.92	103.31
IRF1	16.64	81.50	72.11	340.19
ISG20	2.12	14.98	9.17	62.55
JUNB	17.61	114.45	76.33	477.73
KLF4	13.08	68.53	56.67	286.07
KLHL38	0.70	4.35	3.05	18.16
KRTAP4-12	0.24	1.15	1.02	4.82
LDHAL6B	0.23	1.10	0.99	4.57
LRRTM2	0.12	1.32	0.54	5.51
LTB	0.08	1.03	0.36	4.28
MAFF	6.06	26.56	26.26	110.88
MAGEA2B	0.24	1.75	1.02	7.31
MAK	0.48	1.99	2.07	8.30
MAP3K8	0.58	4.37	2.51	18.26
MRGPRX3	0.07	1.29	0.32	5.37
MSC	2.43	10.91	10.52	45.52

MX1	2.34	14.14	10.13	59.03
MXD1	4.83	21.01	20.92	87.69
NFKBIA	13.69	190.33	59.31	794.51
NFKBIZ	3.45	21.35	14.97	89.11
NOTCH1	6.45	2614.22	27.94	10912.59
NUAK2	3.16	25.80	13.70	107.71
OTUD1	6.78	40.79	29.37	170.28
PAX5	0.10	1.11	0.42	4.64
PNRC1	5.56	22.80	24.12	95.17
PPP1R15A	79.74	369.69	345.58	1543.21
PTGS2	0.20	1.75	0.89	7.29
RASGRP3	0.22	1.76	0.94	7.34
RELB	2.11	13.86	9.14	57.88
RGS2	0.27	1.27	1.18	5.31
RNASE4	1.17	0.00	5.09	0.00
RND1	0.15	1.53	0.67	6.39
RSAD2	49.48	214.06	214.42	893.57
RTP4	2.57	13.60	11.12	56.78
SAA1	0.25	2.93	1.08	12.21
SAA2	0.11	2.34	0.49	9.79
SLC12A7	0.80	5.18	3.47	21.62
SLC8A2	0.09	1.84	0.40	7.67
SMTNL1	0.34	2.21	1.46	9.23
SOD2	12.37	74.14	53.60	309.50
SPTBN5	0.42	2.27	1.80	9.48
STARD5	1.24	5.69	5.37	23.74
TEX29	0.34	1.69	1.48	7.07
TICAM2	1.81	0.43	7.83	1.81
TMEFF1	4.45	0.00	19.28	0.00
TNF	0.04	7.67	0.16	32.00
TNFAIP3	12.48	259.38	54.07	1082.72
TNFSF10	2.04	11.77	8.85	49.12
TNFSF13B	1.41	8.89	6.10	37.10
TNFSF14	0.20	1.86	0.85	7.77
TRAF1	0.22	6.32	0.94	26.40

USP43	0.84	3.47	3.63	14.47
WARS	41.98	196.01	181.92	818.21
XAF1	1.00	7.56	4.32	31.55
XIRP1	0.27	4.15	1.19	17.34
ZBP1	0.30	2.43	1.32	10.16
ZC3H12A	2.92	12.45	12.67	51.95
ZC3HAV1	137.29	563.97	594.98	2354.18
ZC3HAV1L	0.07	1.18	0.31	4.93
ZFP36	8.41	73.73	36.44	307.79

FPKM: Reads Per Kilobase per Million
TPM: Transcripts Per Million
